# Rhabdomyolysis in an HIV cohort: epidemiology, causes and outcomes

**DOI:** 10.1186/s12882-017-0656-9

**Published:** 2017-07-17

**Authors:** Sahar H. Koubar, Michelle M. Estrella, Rugmini Warrier, Richard D. Moore, Gregory M. Lucas, Mohamed G. Atta, Derek M. Fine

**Affiliations:** 1 Department of Medicine/Division of Nephrology, American University of Beirut Medical Center and School of Medicine, Riad El Solh, PO Box 11-0236, Beirut, 1107 2020 Lebanon; 2 Kidney Health Research Collaborative, San Francisco VA Medical Center and University of California, 4150 Clement St., 111A1, San Francisco, California, CA 94121 USA; 3 Lincoln Nephrology & Hypertension, Lincoln, 7441 O St., Suite 304, Nebraska, 68510 USA; 4 Department of Medicine/Division of Infectious Diseases, Johns Hopkins University Hospital and School of Medicine, 1830 E. Monument St., Room 435A, Baltimore, MD 21287 USA; 5 Department of Medicine/Division of Nephrology, Johns Hopkins University Hospital and School of Medicine, 1830 E. Monument Street - Suite 416, Baltimore, MD 21205 USA

**Keywords:** Acute kidney injury, AIDs, Creatinine kinase, HIV, Rhabdomyolysis

## Abstract

**Background:**

The Literature on rhabdomyolysis in the HIV-positive population is sparse and limited. We aimed to explore the incidence, patient characteristics, etiologies and outcomes of rhabdomyolysis in a cohort of HIV-positive patients identified through the Johns Hopkins HIV clinical registry between June 1992 and April 2014.

**Methods:**

A retrospective analysis of 362 HIV-positive patients with non-cardiac CK elevation ≥1000 IU/L was performed. Both inpatients and outpatients were included. Incidence rate and potential etiologies for rhabdomyolysis were ascertained. The development of acute kidney injury (AKI, defined as doubling of serum creatinine), need for dialysis, and death in the setting of rhabdomyolysis were determined. Logistic regression was used to evaluate the association of peak CK level with the development of AKI.

**Results:**

Three hundred sixty two cases of rhabdomyolysis were identified in a cohort of 7079 patients with a 38,382 person years follow-up time. The incidence rate was nine cases per 1000 person-years (95% CI: 8.5–10.5). Infection was the most common etiology followed by compression injury and drug/alcohol use. One-third of cases had multiple potential etiologies. AKI developed in 46% of cases; 20% of which required dialysis. Thirteen percent died during follow-up. After adjustment, AKI was associated with higher CK (OR 2.05 for each 1-log increase in CK [95% CI: 1.40–2.99]), infection (OR 5.48 [95% CI 2.65–11.31]) and higher HIV viral load (OR 1.22 per 1-log increase [95% CI: 1.03–1.45]).

**Conclusion:**

Rhabdomyolysis in the HIV-positive population has many possible causes and is frequently multifactorial. HIV-positive individuals with rhabdomyolysis have a high risk of AKI and mortality.

## Background

Rhabdomyolysis is common in the HIV-positive population, particularly in those with advanced disease [1]. A cohort study from Kaiser Permanente reported a 10-fold higher incidence of rhabdomyolysis among the HIV-positive compared with HIV-negative individuals (265 events/100,000 py versus 26 events/100,000 py; 95% CI: 8.5–12.0; *p* < 0.001) [1]. The spectrum of the illness varies from asymptomatic elevation in muscle breakdown biomarkers to a life-threatening disease causing severe acute kidney injury (AKI), major electrolyte disturbances and death in some cases. Rhabdomyolysis is responsible for 5–25% of all cases of AKI [2] and is associated with death in up to 10% of these cases [3].

Risk factors unique to this population include HIV itself [4, 5], co-infection with HCV [6, 7], medication-related adverse effects, drug-drug interactions [8], and alcohol and illicit drug abuse [9]. Despite the notably higher incidence of rhabdomyolysis among HIV-positive persons, the literature in this patient population is sparse. Studies to date have been relatively small and dependent on diagnostic codes (e.g. ICD-9 codes) [1, 10–16]. To more comprehensively evaluate the risk factors for rhabdomyolysis and its impact on clinical outcomes in the context of HIV infection, we aimed to investigate the incidence, patient characteristics, etiologies and outcomes of rhabdomyolysis in a large cohort of HIV-positive patients cared for at a tertiary care center in Maryland, USA between June 1992 and April 2014.

## Methods

### Study design and population

Our study was a retrospective cohort study of HIV-positive individuals who presented with rhabdomyolysis either as outpatients or inpatients at the Johns Hopkins Hospital. Patients were identified through the review of the laboratory records of those enrolled in Johns Hopkins HIV clinical registry between June 1992 and April 2014. We included individuals aged 18 years or older with a creatinine kinase (CK) level ≥ 1000 IU/L. This level is >5 times upper normal limit at our laboratory (200 by the Roche CK reagent. Indianapolis, IN, 2009) [17] and it has been used to define rhabdomyolysis in prior studies [18, 19]. This cut-off level of CK provides a wide range of levels but excludes mild cases of rhabdomyolysis. Baseline and peak creatinine kinase levels were recorded and the latter was used for analysis. Patients with an elevated CK as a result of myocardial injury (determined by chart review) were excluded from the study. The study was approved by the Johns Hopkins Medicine Institutional Review Board.

### Data collection and classification of causes

Data extraction occurred through extensive review of the inpatient and outpatient electronic medical records of the eligible patients. Several demographic characteristics were extracted including age, sex, hepatitis C virus (HCV) and hepatitis B virus (HBV) serostatus, smoking history, alcohol use, as well as cocaine and heroin use. The latter three were categorized as current, remote or none. Remote status refers to >1 month before the occurrence of rhabdomyolysis. Several laboratory parameters were abstracted including peak CK level, CD4 cell count and HIV-1 viral load. If the latter two were not up to date during the acute episode, the closest values within 3 months were used. We defined a suppressed viral load as <400 copies/ml.

Potential etiologies for rhabdomyolysis in each participant were identified. We divided the causes into 14 categories including compression injury, infection, seizure, alcohol, cocaine, medications, cardiopulmonary arrest, surgery, hypokalemia, HIV-associated myositis, physical exertion, ischemic limb, hypothyroidism, unclear and miscellaneous. Compression injuries encompassed those from immobilization, compartment syndrome or direct trauma. Urine toxicology screens during the episode were reviewed on each patient and cocaine was considered a contributing etiology if the toxicology screen was positive for cocaine. Medications were labeled as an etiology if these medications were considered as such by the treating clinicians and if CK levels normalized with discontinuation of the medication (s). Cardiopulmonary arrest was considered an etiology when chest compressions or defibrillation were performed during resuscitation. Surgery was considered an etiology when CK elevation occurred exclusively in the context of prolonged surgery or surgery on a limb in the absence of other identifiable factors. Hypokalemia was considered a contributing etiology if it was reported as an etiology in the medical records, and the potassium level was <2.8 meq/L. Electromyography reports were reviewed when available. The diagnosis of HIV-associated inflammatory myopathy [20, 21] was based on reported diagnosis, available electromyography or biopsy reports. Miscellaneous causes included heat stroke, neuroleptic malignant syndrome, hypophosphatemia, hypomagnesaemia, hypocalcemia, hypothermia, Amyotrophic Lateral Sclerosis, unknown drug overdose, muscle injection, hyperosmolar hyperglycemic state, muscle spasms, myoclonic jerks, leaky muscle in African American, polyarticular gout and sickle cell crisis [22]. The etiology was labeled as unclear when rhabdomyolysis was not characterized in the medical record and no etiologies were identified by review of the historical documents, laboratory values and follow-up visits. For the eight patients who had recurrent episodes of rhabdomyolysis, only the first episode was included in the analysis.

### Outcomes

Patients were treated as inpatients or outpatients. The primary outcomes of interest were development of AKI and the need for renal replacement therapy (RRT). Serum creatinine levels at baseline, peak and recovery (the lowest within 1 month after the episode of rhabdomyolysis) were recorded. AKI was defined as per KDIGO guidelines defining stage two AKI (doubling of baseline serum creatinine during the episode of rhabdomyolysis). We also performed a sensitivity analysis defining AKI as a rise in serum creatinine of 0.3 mg/dl or greater from baseline, which corresponds to stage I AKI per KDIGO guidelines [23]. Our secondary outcomes were duration of hospital stay and mortality.

### Statistical analysis

Statistical analysis was performed using Stata version 13 (StataCorp College Station, Texas). We categorized patients into three groups according to their peak CK level: 1) low (1000–5000 IU/L); 2) intermediate (>5000 and ≤10,000 IU/L); and 3) high (>10,000 IU/L). Comparisons between the three categories were performed using chi-square for categorical variables and analysis of variance for continuous variables. Univariable and multivariable logistic regression models were constructed to evaluate the association between peak CK level and AKI. The multivariable model was adjusted for potential confounders of this association and included age, gender, HIV-1 RNA level, and presence or absence of sepsis. Patients with end-stage renal disease (ESRD) were excluded from the analysis of AKI (*n* = 26). Ordered logistic regression was used to assess the correlation between CD4 count and different CK categories.

## Results

### Incidence of Rhabdomyolysis

The HIV Clinical registry consisted of 7079 patients with 38,382 person-years of follow-up during the study period of June 1992 to April 2014. The study spanned the pre and post-HAART era. Of 413 patients aged 18 years and older identified as having a CK ≥1000 IU/L, 51 were excluded. Of those excluded, 18 had an elevated CK of cardiac origin, and 33 had no clinical data besides their laboratory data. Therefore, 362 patients were included in this analysis. The overall incidence rate of rhabdomyolysis defined by a CK level ≥ 1000 IU was 943 cases per 100,000 person-years.

### Patient characteristics

Table 1 displays the sociodemographic and clinical characteristics of the study cohort. Sixty patients experienced rhabdomyolysis in the pre-HAART era (1992–1995) and 302 in the post-HAART era (1996–2014). There were 281 outpatients and 81 inpatients. In comparison to the whole HIV registry, the HIV cohort with rhabdomyolysis had more males (76% versus 67%), more black race (88% versus 76%), and higher rates of HCV infection (57% versus 46%) and HBV infection (11% versus 9%).

**Table 1:** Patient characteristics by CK category

Characteristics	Overall (*N* = 362)	CK 1000- < 5000 (*n* = 284)	CK 5000–10,000 (*n* = 33)	CK >10,000 (*n* = 45)	*P*-value
Median Age (y)	45.8 (±9.6)	47	43	42	0.39
Black (%)	88	88	85	93	0.47
Males (%)	76	77	76	67	0.29
HCV positive, n (%)	205 (57)	164 (58)	21 (64)	20 (44)	0.17
HBV positive, n (%)	40 (11)	30 (11)	2 (6)	8 (18)	0.24
Current Smoker, n (%)	240 (66)	187 (66)	21 (64)	32 (71)	0.59
Current Alcohol Use, n (%)	92 (25)	70 (25)	9 (27)	13 (29)	0.82
Current Cocaine Use, n (%)	135 (37)	96 (34)	15 (45)	24 (53)	0.19
Current Heroin Use, n (%)	123 (34)	86 (30)	15 (45)	22 (49)	0.18
No. of possible causes of rhabdomyolysis					0.003
1(%)	60	62	58	47	
2(%)	27	25	30	40	
3(%)	5.5	4	9	11	
4(%)	0.3	0	3	0	
Unclear causes (%)	7	8.5	0	2	
Median Viral Load, copies/ml	20,580	19,371	12,514	43,571	0.69
Median CD4 count, cells/ml	175	192	95	187	0.04

CK values in IU/L

### Etiologies of Rhabdomyolysis

A higher peak CK level was associated with a greater number of potential etiologies underlying the rhabdomyolysis. Two factors were present in 25%, 30% and 40% of the cases in the low, intermediate and high CK level categories, respectively. Three factors were present in 4%, 9% and 11% of the cases in the low, intermediate and high CK level categories, respectively (Table 1).

Table 2 summarizes the frequencies of the contributing etiologies for rhabdomyolysis. Infection was the most common etiology encountered (39% of cases). Two concurrent infections were present in 24% of cases, while three concurrent infections were present in 3.5%.

**Table 2:** Etiologic factors underlying development of rhabdomyolysis in the total HIV cohort and according to viral load^a^

Etiology	Overall (*n* = 362)	Viral load <400 copies/ml (*n* = 71)	Viral Load ≥400 copies/ml (*n* = 291)	*p*-value
Infection	141 (39%)	17 (24%)	124 (43%)	0.004
Pneumonia	75 (21%)	7 (10%)	68 (23%)	
Bacteremia	26 (7%)	4 (6%)	22 (8%)	
Viral Illness	25 (7%)	3 (4%)	22 (8%)	
Pyelonephritis	8	2	6	
Endocarditis	7	0	7	
Fungemia	7	2	5	
Acute antiretroviral illness	6	0	6	
Abscess	6	1	5	
Line Infection	5	1	4	
Necrotizing Fasciitis	5	0	5	
Sinusitis	5	1	4	
Meningitis	4	0	4	
Others^b^	12	2	10	
Trauma/compression	82 (23%)	25%	22%	0.544
Cocaine	76 (21%)	11%	23%	0.025
Seizure	26 (7%)	7%	7%	0.959
Alcohol	24 (6.6%)	6%	7%	0.707
Medications	22 (6%)	17%	4%	<0.001
Cardiopulmonary arrest	20 (6%)	6%	6%	0.964
Surgery	16	6%	4%	0.579
Hypokalemia	12	4%	3%	0.633
HIV Associated Myositis	11	1%	3%	0.372
Physical Exertion	8	1%	2%	0.608
Ischemic Limb	8	4%	2%	0.198
Hypothyroidism	2	1%	1%	0.484
Unclear	25 (7%)	13%	6%	0.032
Miscellaneous	25 (7%)	10%	6%	0.470

^a^Total percentages greater than 100% because 33% of patients had multiple etiologies

^b^Others include appendicitis, cholecystitis, spontaneous bacterial peritonitis, clostridium difficile colitis, osteomyelitis and proctitis

Table 2 also shows the difference in etiologies by viral load (VL). Not surprisingly, those with detectable viral load were more likely to have an infectious etiology (*p* 0.004) while those with suppressed viral load were more likely to have medications underlying the cause of their rhabdomyolysis (*p* < 0.001). Table 3 shows the different medications responsible for rhabdomyolysis individually or secondary to drug-drug interactions.

**Table 3:** List of drugs suspected as the cause of rhabdomyolysis in HIV-positive patients

Drug	No. of events (22)
Statin (HMG-CoA reductase inhibitor)	12
Monotherapy	8
Fenofibrate interaction	2
Ritonavir interaction (simvastatin)	1
Raltegravir + Atorvastatin	1
Zidovudine	3
Raltegravir	2
Abacavir	1
Mirtazapine	1
Colchicine	1
Rifampicin	1
Testosterone	1

### Association of CK level with AKI

Overall, AKI developed in 46% of patients (Table 4). Higher CK levels were associated with AKI; AKI developed in 42% of patients in the lower CK category, 58% in the intermediate CK category and 64% in the higher CK category (*P* = 0.01). Among those with AKI, 20% required RRT.

**Table 4:** Distribution of AKI in the total HIV cohort and according to the CK level category

Total Cohort		CK Categories			
	*N*=336^a^	1000- < 5000 IU/L (*n* = 261)	5000–10,000 IU/L (*n* = 30)	>10,000 IU/L (*n* = 45)	*P*-value
AKI	156/336 (46%)	110 (42%)	17 (57%)	29 (65%)	0.011
RRT	31/336 (9%)	22 (8%)	2 (7%)	7 (16%)	0.52
Death	46/362^b^ (13%)	31/284 (11%)	7/33 (21%)	8/45 (18%)	0.024

^a^ESRD (*n* = 26) were excluded from the analysis

^b^Whole Cohort was used for analysis of mortality

When evaluated continuously, each 1-log higher CK level was associated with a 2.05-fold increased odds of AKI (95% CI: 1.40–2.99). Other factors associated with AKI included presence of infection (OR = 5.48 [95% CI 2.65–11.31]) and higher HIV viral load (OR 1.22 [95% CI: 1.03–1.45]). This association of CK level with the development of AKI remained significant after adjusting for age, sex, viral load, CD4 cell count, and presence of infection (Table 5). This association also remained significant among the subset of patients in the post-HAART era, but was not significant among patients in the pre-HAART era.

**Table 5:** Adjusted association of various predictors with odds of acute kidney injury

	Odds Ratio	95% CI	*P* value
CK level, per 1-log higher	2.05	1.40–2.99	<0.01
Infection vs. no infection	5.48	2.65–11.31	<0.01
HIV viral load, per 1-log higher	1.22	1.03–1.45	0.01
CD4 count, per 100 cells/ml higher	0.98	0.85–1.13	0.80
Age, per 10 years older	0.71	0.46–1.08	0.11
Black vs. non-black	0.92	0.29–2.90	0.88
Male vs. female	0.50	0.62–2.81	0.46

When we analyzed the data based on the absolute increase in serum creatinine by ≥0.3 mg/dl from baseline, the incidence of AKI increased to 62%. Similar to the primary analyses, each 1-log higher CK level was associated with 2.11-fold higher odds of AKI (95% CI: 1.4–3.2).

### Association of CD4 count with CK categories

Higher CD4 counts were associated with lower CK levels. For each one unit increase in CD4 count, the odds ratio of being in a CK category ≥1000 IU/L is 0.99. However, this inverse correlation did not reach statistical significance (*p* value 0.99; 95% CI 0.99–1.00).

### Hospitalization

The median duration of stay for those 81 patients admitted to the hospital was 5 days (IQR 2–13 days); 9 days (IQR 3–17 days) for those with AKI versus 4 days (IQR 1–8 days) for those without AKI (*p* < 0.01). The median hospital stay was 5 days (IQR1–12 days) for the lower CK category, 4 days (IQR 2–12 days) for the intermediate CK category and 8 days (IQR 4–15) for the higher CK category. There was a trend towards increased hospital stay with increased blood CK level but it did not reach statistical significance (OR 1.7; 95% CI 0.8–3.5; *P* value 0.17).

### Mortality

Overall, death occurred in 13% of patients. Of those who developed AKI, 39 patients (25%) died compared to 3 patients (1.7%) in those without AKI (*P* < 0.01). (Table 4).

## Discussion

To the best of our knowledge, this study represents the largest to date evaluating causes and outcomes of rhabdomyolysis in an HIV-positive population. Our study showed a relatively high incidence of rhabdomyolysis as defined by CK ≥1000 IU/L. Infection, compression injury and drug/alcohol use were the most common etiologies present in these patients. In contrast to other studies, multiple concurrent potential etiologies were frequently identified in this population.

These processes lead to muscle necrosis through direct sarcolemmic injury or by interfering with the oxidative or glycolytic energy pathway, ultimately leading to lethal intra-sarcoplasmic calcium overload, ATP depletion and subsequently muscle cell death. The incidence of AKI was very high in this patient population at 46%, though in large part a result of an infection associated with the rhabdomyolysis.

The incidence of rhabdomyolysis in our HIV population was 943 per 100,000 person-years. Reported data from Kaiser Permanente on HIV-positive individuals showed an incidence rate of 265 per 100,000 person-years [1]. Studies assessing incidence rates of rhabdomyolysis in the general population have specified those based on particular etiologies. The incidence rate of rhabdomyolysis secondary to lipid-lowering drugs ranged between 2.46 to 4.4 cases/100,000 person-years with statin monotherapy and up to 37–60 cases/100,000 person-years with statin/fibrate combination [24, 25]. The latter is comparable to the incidence rate of rhabdomyolysis associated with statins in our population which is around 55 per 100,000 person years. The incidence rate was 22.2 cases per 100,000 person-years in military trainees with exertional rhabdomyolysis [26]. Therefore, compared to the prior studies, the incidence rate in our population was significantly higher, suggesting higher risk of rhabdomyolysis in the HIV-positive population. This is likely related to the higher risk of infection in this population, higher rates of drug exposures (illicit and non-illicit), and possibly higher risk of drug-drug interactions. In addition, differences among studies with regards to incidence could also be due to differing thresholds used to define rhabdomyolysis. Similar to other studies of rhabdomyolysis, the majority of patients were men. This likely reflects their increased muscle mass and possible increased frequency of exposure to risk factors.

More than one potential contributory factor was present in 33% of patients. Multiple risk factors resulted in more severe injury and higher CK levels. This is in contrast to the study by Melli et al. which did not show a correlation between increasing number of risk factors and higher CK levels [27].

Infection was the most frequently encountered etiology among the HIV-positive population, but it was associated with milder CK elevations. Seventy-five percent of patients with infection had a CK level between 1000 and 5000 IU/L. Sepsis as a major cause for rhabdomyolysis in the HIV-positive population was identified in six out of a series of seven patients by Joshi and Liu [16] and in seven out of a series of 20 by Chariot et al. [15]. Pneumonia was the most common etiology identified in our population; consistent with what has been demonstrated in a study of 52 patients by Blanco et al. [28] who assessed infectious versus non-infectious causes of rhabdomyolysis. Respiratory infections constituted 38% of the infectious etiologies. This could reflect the fact that pneumonia accounts for most infectious admissions to the hospital [29], but also raises the question of whether hypoxia secondary to pneumonia further potentiates muscle cell necrosis. Two or more concomitant infections – viral, bacterial or fungal - were seen in 27.5% of our patients. This likely speaks to the immunocompromised status of this particular set of patients and their vulnerability to infectious complications.

Compression injury was the second most common etiology in our population. We attribute this to the increased prevalence of poly-substance abuse in our study population, with associated increased risk for immobilization and consequent pressure on muscle groups. It might also reflect the socioeconomic background of this population that puts them at increased risk for traumatic injury [30, 31].

HIV-associated inflammatory myositis was present in 11 cases (3%). All of these were in the post-HAART era. Melli et al. described two cases of HIV associated myositis over a 9-year period [27]. The increased incidence in our study might imply an increased recognition of this disease entity or, possibly, poor adherence of our study cohort with their antiretroviral therapy, since 10 of 11 had viral load >400 copies per ml.

Statins were responsible for >50% of medication-induced myotoxicity, whereas in other reports, antipsychotic medications were the leading etiology of medication-induced rhabdomyolysis [27], and the cause of recurrent rhabdomyolysis in 10% of patients [28]. Our results are likely explained by the increased prevalence of dyslipidemia and atherosclerotic disease associated with cART and thus the increased frequency of statin use. In addition, drug-drug interactions between statins (those metabolized through the cytochrome P450 3A4 [CYP3A4] enzyme system: simvastatin, lovastatin, and atorvastatin) and protease inhibitors, particularly the powerful CYP3A4 inhibitors ritonavir or cobicistat, are well known [8]. Previous studies have reported myotoxic medications to be responsible for 11% of cases [27]. The lower percentage in our study likely reflects disproportionate rates of infection, drug and alcohol use in this cohort, and possibly reflects medication non-adherence in our patient population.

AKI developed in 46% of our patients. Comparison of the incidence of rhabdomyolysis associated AKI in the HIV-positive population to the general population is difficult due to varying definitions of AKI used and different cutoffs for CK level to define rhabdomyolysis; the reported incidence in the general population ranges from 13 to 46%, so that rhabdomyolysis-associated AKI in this particular HIV-positive population is at least at the higher end of the spectrum.

Higher CK levels were associated with increased incidence of AKI. This is consistent with findings from several other studies [3, 32]. Not surprisingly, the need for RRT was also higher in the higher CK groups, though the difference did not reach statistical significance between the three different CK categories. This is likely secondary to the small proportion that needed dialysis in each group.

The mortality rate was higher in the intermediate and high CK groups at 21% and 18% respectively, compared with 11% in the low CK group. We attributed this high mortality to the increased proportion of patients with infection in those groups (55% and 38% respectively|), where CK elevations were likely related to the infection itself. CK elevation with infection reflects muscle ischemia from a prolonged hypo-perfusion which also results in ischemic renal injury, and intracellular acidosis leads to ATP depletion and increased risk of muscle necrosis. In addition, mild elevations of CK have been reported in febrile patients [33].

As was shown previously by Ward et al. [18], the mortality of hospitalized patients with a CK level > 1000 IU was higher in those who developed AKI versus those who did not develop AKI. This was true in our population as well. In addition, rhabdomyolysis in this HIV population was associated with a 1.5–4 fold increase in the rate of death than the general population (13% in this study compared to 3.4–10% in the general population reported in prior literature) [27].

Our study has several limitations. It is a retrospective study where data collection was based on chart reviews. Nonetheless, we did not solely rely on reported diagnosis in discharge summaries. Follow up clinic notes, response to interventions/treatments, laboratory values, toxicology screens, electromyographies and biopsies were reviewed on each patient when available. Also, this is a single urban center study with a predominantly African American population and many of those studied had virologically uncontrolled HIV disease. As noted above the study also spanned the different treatment eras of HIV care. These factors limit its generalizability to other cohorts of different races and ethnicities and less severe HIV disease.

On the other hand, this is the largest HIV cohort studied regarding the epidemiology, causes and outcomes of rhabdomyolysis. Patients were selected based on their lab results rather than on ICD 9 codes, which may miss some mild cases of rhabdomyolysis. To our knowledge, there are 2 other studies that assessed rhabdomyolysis in the context of HIV infection. In a series of 20 hospitalized HIV-positive patients in France [15], rhabdomyolysis was responsible for 10% of cases of biopsy-proven AKI. In another series of seven patients, three patients developed AKI, and one of those patients died [15, 16].

## Conclusion

Infection appears to be the most common etiology of rhabdomyolysis (CK > 1000 IU/L) in HIV-positive patients. One third of cases are potentially multifactorial. Both increasing CK level and infection are independently associated with development of AKI in this cohort. Rhabdomyolysis in HIV appears to be associated with an increase in mortality compared with the general population. This likely reflects a vulnerable population at increased risk of sepsis, where elevated CK might serve as a marker of poor outcome in those patients.

## Abbreviations

AKI: Acute kidney injury
ATP: Adenosine triphosphate
cART: Combination anti-retroviral therapy
CK: Creatine kinase
HAART: Highly active anti-retroviral therapy
HBV: Hepatitis B virus
HCV: Hepatitis c virus
HIV: Human immunodeficiency virus
IQR: Inter-quartile range
OR: Odds ratio
RRT: Renal replacement therapy
VL: Viral load
